# Genetic Determinants of the Re-Emergence of Arboviral Diseases

**DOI:** 10.3390/v11020150

**Published:** 2019-02-12

**Authors:** Harshada Ketkar, Daniella Herman, Penghua Wang

**Affiliations:** 1 Department of Microbiology & Immunology, New York Medical College, Valhalla, NY 10595, USA; Harshada_Ketkar@nymc.edu (H.K.); dherman@nymc.edu (D.H.); 2 Department of Immunology, School of Medicine, The University of Connecticut Health Center, Farmington, CT 06030, USA

**Keywords:** Arbovirus, host, genetic, evolution, vector, re-emergence

## Abstract

Mosquito-borne diseases constitute a large portion of infectious diseases, causing more than 700,000 deaths annually. Mosquito-transmitted viruses, such as yellow fever, dengue, West Nile, chikungunya, and Zika viruses, have re-emerged recently and remain a public health threat worldwide. Global climate change, rapid urbanization, burgeoning international travel, expansion of mosquito populations, vector competence, and host and viral genetics may all together contribute to the re-emergence of arboviruses. In this brief review, we summarize the host and viral genetic determinants that may enhance infectivity in the host, viral fitness in mosquitoes and viral transmission by mosquitoes.

## 1. Introduction

Humans have known about arboviruses since the 15th century when yellow fever was first described as devastating the human populations in Africa, the Americas, and Europe [1]. From the 17th through 20th centuries, vector-borne diseases were the biggest cause of human diseases and deaths than all other causes combined. In the 20th century, prevention and control were achieved by managing the vectors through insecticides and removal of breeding sites. By the 1960s, the efforts had proven to be successful and these diseases that had been considered a public health problem were no longer causing devastation on a grand scale [2]. However, the past several decades have seen the re-emergence of arboviruses in areas outside of Africa. Today, infectious diseases are the second-most common cause of death worldwide and the first in developing countries, causing as many as 15 million deaths each year according to the World Health Organization.

The most common arboviruses that have caused recent epidemics are the flaviviruses, including yellow fever, dengue, West Nile, and Zika, and the Toga viruses, which include Chikungunya. The Centers for Diseases Control and Prevention (CDC) of the United States estimates that yellow fever virus (YFV) causes 200,000 cases and 30,000 deaths globally each year, primarily in Africa [3]. Some of these urban epidemics may have been caused by globalization and travel, which makes it easier to transmit YFV across borders [4]. Dengue virus (DENV) distribution has been growing for the past 40 years and now it is expected to infect 390 million people annually [5]. The emergent dengue disease epidemic in the Americas in the 1990s was found to be similar in epidemic potential as occurred in Southeast Asia 30 years before that epidemic [6]. West Nile Virus (WNV) reached the Western Hemisphere in 1999 and quickly became one of the most widely circulating arboviruses worldwide [7]. By November 2018, as many as 1500 cases of WNV infection were reported in the European Union, exceeding the total number of infections in the previous five years [8]. Chikungunya virus (CHIKV) reached the Americas in late 2013 and quickly spread to the surrounding countries. By 2017, it had caused more than 1.8 million suspected cases in 44 different countries [9]. Zika virus (ZIKV) has caused one of the most recent epidemics to be considered a public health crisis. ZIKV was present for decades prior to 2007 outbreaks in Asia, and was introduced to the Americas by 2014. Over half a million autochthonous and 3700 microcephaly cases had been reported by January 2018 according to the Pan American Health Organization. In the United States and its territories, over 40,000 symptomatic ZIKV infections were reported from 2015 through to October 2018 [10]. 

**Figure 1:**
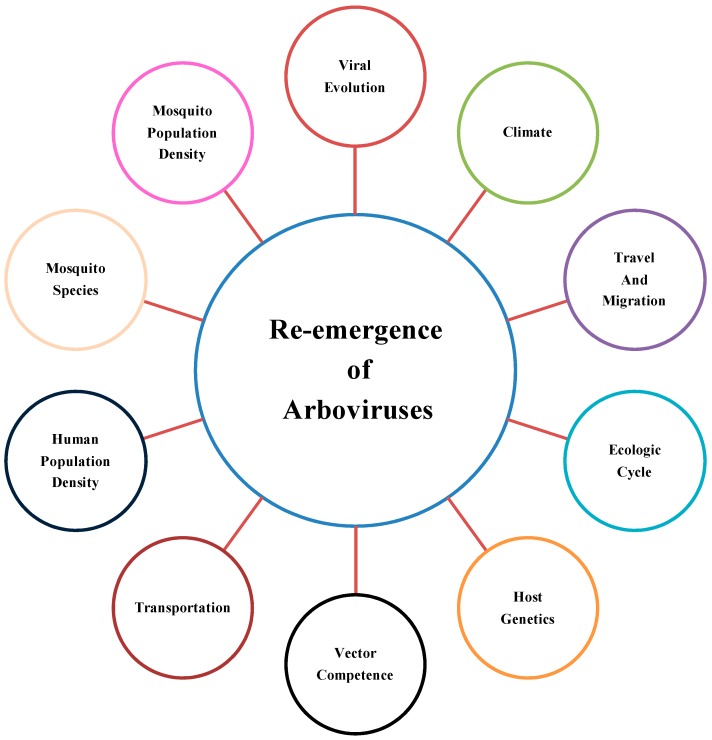
Factors contributing to the re-emergence of arboviruses.

There are a number of factors that may have contributed to the recent reemergence and spread of arboviral diseases. Increased episodes of DENV infections are rooted in global population growth, urbanization, lack of mosquito control measures, increased air travel, and decay in public health [11]. The 1979 DENV3 outbreak in Central Java, Indonesia, was characterized by low viremia. The mild illness in human was proposed to be associated with the maintenance of different endemic strains, which could give rise or co-circulate with the epidemic strains [12]. Similarly, the 2007 dengue outbreak in Singapore showed serotype change and clade replacement, which empowered co-circulation of several genotypes [13]. An additional factor considered to contribute is seroepidemiology. Most cases of dengue resurgence in Singapore after 1986 emerged in the young adult population [14]. Another study claimed that the combination of lowered herd immunity and the failure of vector control opened the door to re-emergence [15]. The strategy suggested for the prevention of disease is an integrated regional approach incorporating efficient surveillance, emergency response, and case management, as all these factors contribute to a persistent disease cycle [16]. Severity of the disease was also found to be greater with a prolonged interval between primary and secondary dengue infection in island outbreaks [17]. During 2005 Dengue outbreak in Singapore ecological and immunological factors were indicated to be responsible for epidemics [18]. Additional determinants of the re-emergence include travel and transport, environmental factors, ecological cycles of vectors, host genetic factors, viral evolution, human and mosquito population density, mosquito species, and vector competence (Figure 1). In this review, we will discuss the host and viral genetic variations associated with increased arboviral pathogenicity, infectivity, vector fitness, transmissibility, and epidemic potential. 

## 2. Human Genetic Determinants of Viral Pathogenicity

Genetic diversity underlies individual differences in human disease pathogenesis and severity. Not surprisingly, the outcomes of arboviral infection are associated with many genetic mutations and single nucleotide polymorphisms (SNPs), the majority of which are related to immune pathways (Table 1). The type I interferon (IFN) response is induced rapidly following a viral infection and is an essential early antiviral mechanism. SNPs in the genes of the type I IFN pathway influence the severity of arboviral infection in humans. SNPs in an interferon stimulated gene (ISG) for 2′-5′-oligoadenylate synthetase (OAS), an enzyme involved in the innate immune response to viral infection by destroying viral RNA, are likely associated with severe infections. In mice, a nonsense mutation, C820T, in the exon 4 of the *Oas1b* gene produced a truncated protein that inhibits the enzymatic activity of Oas1b. This allowed for greater replication of WNV in neurons with the mutated enzyme and enhanced viral infection [19,20]. A synonymous SNP in human *OASL* exon 2 (rs3213545) that leads to a similar dominant negative OASL isozyme was more frequent in hospitalized WNV patients than control subjects [21]. A splicing variant (rs10774671) and an intron variant (rs34137742) of *OAS1* were associated with severe WNV infection [21,22,23]. Additionally, a variant of OAS3, OAS_R381, was less effective in activating the RNase used to decimate viral RNA. This produced decreased antiviral activity toward DENV-2 and manifested as a more severe dengue infection [24]. SNPs in interferon regulatory transcription factor (IRF3), an important transcription factor for type I IFNs, and myxoirus resistance 1 (MX1), an interferon-induced dynamin-like guanosine triphosphate (GTP)ase, were correlated with symptomatic WNV infection; however, the mechanism is not well understood [23].

SNPs in viral recognition receptors that elicit immune responses or aid in viral entry into host cells may also contribute to arboviral pathogenesis. Infection with CHIKV activates Toll-like receptors (TLR) that initiate the innate immune response, including inflammatory cytokines and type I IFNs. Three SNPs in human TLR-7 (rs179010, rs5741880, rs3853839) and one in TLR-8 (rs3764879) were potentially associated with increased disease susceptibility, as well as the enhanced likelihood of developing fever, joint pains, and rash in those infected with CHIKV [25]. The SNP in C-type lectin CLEC5A (rs1285933) might render humans more susceptible to severe dengue diseases [26,27], and this was substantiated by studies in Clec5a knockout out mouse [28]. The rs4804803 SNP (G) in the cluster of differentiation 209 (CD209) [(encodes Dendritic Cell-Specific Intercellular adhesion molecule-3-Grabbing Non-integrin (DC-SIGN)) promoter region could contribute to the pathogenesis of dengue hemorrhagic fever (DHF) in Thai and Taiwanese populations [29,30]. The SNP (A to G in rs1801274 changes histidine to arginine) in immunoglobulin heavy chain receptor FcγRIIa was correlated to more severe dengue infection in Pakistani [31] and Cuban [32] populations. However, contrasting results were reported for the same SNPs in CD209 and FcγRIIa in Mexicans [33]. These studies highlight the importance to interpret human population genetics results in conjunction with the specific ethnic background. 

Another study performed on blood donations in Guadeloupe and Martinique found increased susceptibility to Chikungunya infection in people with blood group A, Rh positive. This link between blood groups and CHIKV susceptibility may be related to several factors that are not well understood, such as different capacities to eliminate viruses by innate immune responses or being more prone to mosquito bites [34]. Mutations in Musashi (MSI1) proteins, important for progenitor cell growth and differentiation, may also be related to brain abnormalities caused by ZIKV infection. Polymorphisms in the 3′ UTR of these proteins may disrupt protein binding and facilitate viral replication. Malfunction of MSI1 led to deregulation of expression of factors required for normal neural stem cell function and embryonic brain pathology [35].

Another set of mutations that affect the host susceptibility to viruses and severity of infection are those in the human leukocyte antigen (HLA) alleles, which encode the major histocompatibility complex (MHC) proteins of the adaptive immune system. This association may be related to the ability of class I and class II alleles to provoke a strong CD4^+^ or CD8^+^ T-cell response. Weaker responses were correlated with susceptibility to symptomatic disease [36]. A study on WNV in Greece found that the patients with DQA1*01:02 had increased susceptibility to infection due to deficient MHC-II. This was also true for patients in Brazil with *HLA-DQ*1 [37]. HLA-A*68 and C*08 could be associated with severe WNV infection in Caucasians in Canada and the United States [38]. In Vietnam, it was found that children with HLA-A*24 were at an increased risk of developing DHF or dengue shock syndrome (DSS) [39,40]. The researchers theorized that this may be due to a heightened CD8^+^ response that could affect vascular permeability and cause tissue damage by viral mimicry of host proteins [40]. In a larger study on Thai patients, researchers discovered that HLA-A*0207 and HLA-B*51 were associated with severe DHF in patients with secondary DENV-1 and DENV-2 infections, while a number of other genotypes were protective against developing severe symptoms during the secondary infections [41]. In a study on the Cuban population, researchers found that polymorphisms in Class I, HLA-A*31 and HLA-B*15, were associated with symptomatic dengue infection [42]. Genotypes HLA-DQB1*0302 and -DQB1*0202 were positively linked to DHF and DF susceptibility, respectively [43]. Sri Lankans with HLA-A*31 and HLA-DRB1*08 might be more susceptible to DSS, during the secondary infection, and people with HLA-A*24 and HLA-DRB1*12 were more likely to have DHF during the primary dengue infection [44].

SNPs in gene encoding cytokines and chemokines also contribute to infection outcomes. There is an increased likelihood of symptomatic WNV infection in people with a C-C chemokine receptor 5 (CCR5)∆32 mutation. CCR5 promotes transport of leukocytes into the infected brain in order to contain and clear the virus. In the CCR5∆32 carriers, WNV replication could not be controlled in the brain, leading to symptomatic infection [45]. *TNFA* (-308, rs1800629) A allele and *IL10* (-1082/-819/-592) ACC/ATA haplotype were significantly associated with DHF in Cubans [46], Sri Lankans [47], and Venezuelans [48,49]. *TNFA* -238A allele and -238GA genotype were associated with DHF/DSS in Malaysians [50]. The homozygous form of an α-tryptase allele of *TPSAB1* was associated with DSS in the Vietnamese and Filipino populations [51], SNPs in phospholipase C epsilon 1 *PLCE1* (rs3765524 and rs3740360) with DSS in the Thai [52] and Vietnamese populations [53]. 

## 3. Viral Genetic Determinants of Infectivity in the Host

A significant cause of viral re-emergence is the evolution and genetic mutations of the viruses that make them more virulent and allow for widespread epidemics (Table 2). RNA viruses acquire genetic diversity because of error-prone RNA-dependent RNA polymerase and a large population of infected vectors and hosts [54]. This fosters high mutation rates and recombination that support adaption to changes in the environment or host immunity [55]. For instance, by the Bayesian method, the overall evolutionary rate of DENV was 7.6 × 10^−4^ substitution/site/year [56]. The role of viral genetics in DHF is supported by the fact that two distinct genetic makeups led to the difference in disease severity in DENV3, subtype III [57]. A new method was developed during 1981 for virological surveillance, which included the use of mosquito C6/36 cell culture and specific anti-dengue monoclonal antibody for isolation and identification techniques. It allowed rapid monitoring of circulating DENV strain and prediction of epidemic potential [58]. An epidemiological study carried out during the 1989 DHF outbreak in Sri Lanka confirmed that the emergence was not correlated with the transmission or the distribution of DENV serotypes. It was proposed that the viral strain and the small genetic changes caused the intransigent and active disease cycle [59]. Similar observations were found in Tonga DENV2 outbreak in 1975. The severe disease pathogenesis was not related to viral profusion, host susceptibility or vector competence, but was suggested due to viral virulence [60].

Few studies were carried out to deduce the role of viral molecular evolution in driving the epidemics. The phenotypic effect of genetic substitutions depends on the gene function and the advantage of the substitution for the virus in the vector or host. DENV4 lineage turnover in 1998 Puerto Rican epidemic was distinguished by three non-conservative amino acids changes in NS2A [61]. The Puerto Rican DENV2 subtype IIIb was replacing the subtype V in the outbreak in 1980. It was associated with molecular changes in the envelope protein. Amino acids were found to be under positive selection, namely E91 and E129 substitutions were conservative; E-131 was non-conservative; E491 was conservative; while E359 changed from T to A [62]. In Managua, Nicaragua, it was found that the severity of dengue infection was associated with the replacement of predecessor DENV2 NI-1 clade by NI-2B clade. The circulating NI-1 clade was known to be of Asian/American origin with substitutions in capsid R97K, NS1-K94R, and NS3 P245T. A single mutation of N245S in NS4B led NI-1 evolution into the NI-2 clade. Further five mutations namely-M492V in the envelope, L279F in NS1, and K200Q, T290I, and R401K in NS5 were shown to drive NI-2 into a more infective strain, NI-2B [63]. During 2001–2002, severe DHF cases were increased in DENV2 outbreak in Taiwan. Genome analysis between two outbreaks within a year revealed five nucleotide changes in E, NS1, NS4A, and NS 5 gene, suggesting molecular evolution of the virus [64]. However, some spontaneous mutations may make DENV less advantageous in the host. One study investigating phylogenetic events that occurred in a DENV-2 1971 South Pacific outbreak revealed that substitutions in prM and non-structural genes NS2A and NS4A led to attenuation of infection [65].

Subgenomic flaviviral RNA fragment (sfRNA), an extension of the 3′-UTR of flaviviral genomes, accumulates during replication and plays an essential role in replication and pathogenesis in both the host and vector [66]. The PR-2B DENV-2 clade, which was fitter than PR-1 DENV2 and caused a severe epidemic in Puerto Rico in 1994, produced more sfRNA than genomic RNA during replication in the human host. sfRNA suppressed the type I IFN response by inhibiting TRIM-25, an E3 ubiquitination ligase, leading to epidemiological fitness [67]. 

Variations in epidemic potential are also attributable to a virus’ capacity to interfere with the host immune system. TSV01 strain of DENV2 activated a robust type I IFN response; while NGS strain suppressed it via signal transducer and activator of transcription 1 (STAT1) and STAT2 [68]. In Brazil, the L6 lineage of DENV1 remained persistently circulating even after the introduction of a more fitting L1 lineage into human and vector. The reason could lie in moderate immune stimulation of the B cell and T cell responses by L6, which allowed for increased systemic replication and viremia [69]. 

Some genetic changes can influence virulence, making the virus more infective or cause worse symptoms. Mutations in the domain III of the ZIKV envelope protein may be an important factor in viral fitness as V603I and D679E substitutions were only seen in the recent epidemic ZIKV strains but not in pre-epidemic strains [70]. Mutation in the viral precursor membrane protein (prM) has been shown to intensify symptoms of ZIKV infection. The S139N substitution arose before the 2013 outbreak on the French Polynesia Island and has been consistently present in the subsequently epidemic American strains. The mutant prM might increase infectivity in neural progenitor cells and promote apoptosis, possibly producing the microcephaly and other pathologies seen in pregnant women during the most recent epidemic [71].

## 4. Viral Genetic Determinants of Vector Fitness and Transmissibility

Mutations have been discovered to assist an arbovirus in its acquisition by a vector as well as adapting to a new one. The nonstructural protein (NS) 1 of a number of flaviviruses including YFV, WNV, Japanese encephalitis, tick-borne encephalitis, DENV, and ZIKV is secreted out of host cells and is present in the host blood [72,73,74,75,76] in a large amount [77] during acute infection. In addition to aiding in flaviviral pathogenesis in the host, NS1 when acquired together with virions enhances viral infectivity in mosquitoes by overcoming the gut immune barrier. This feature of NS1 can increase the chance of viral acquisition by mosquitoes during a short viremic phase and viral prevalence in nature. This might be a survival strategy that arboviruses have evolved to cycle efficiently between two strikingly different host environments [76]. Mutations in the viral genome that influence NS1 secretability may thus impact viral transmission from the vector to host and/or vice versa. Indeed, an alanine to valine substitution at the 188^th^ position (A188V) in NS1 of the recent American ZIKV isolates enhanced viral infectivity and prevalence in mosquitos. This mutation made NS1 highly secretable in the mammalian host, increasing ZIKV transmissibility from the host to vector. This could partly contribute to recent epidemics in South America [78].

*Aedes Aegypti* was a primary vector for CHIKV in India and other countries during the 2006 to 2010 epidemics. The K211E in the envelope protein E1 and V264A in E2 were reported to increase CHIKV adaptation to *Ae. Aegypti* [79,80]. In the recent 2016 CHIKV outbreak in Brazil, researchers discovered two new substitutions in the virus, K211T in E1 and V156A in E2, which could enhance mosquito fitness, allowing the outbreak to become an epidemic as they did for a similar epidemic in India in 2006 [81]. In CHIKV, a change at the position 226 of E1 protein from “A” version (in the strains before 2005) to “V” version (in 90% strains after 2005 epidemics in the Indian Ocean) rendered the virus independent of cholesterol to infect host cells. This is particularly critical for CHIKV prevalence in *Aedes albopictus* mosquitoes that often have insufficient cholesterol to support productive viral replication [82]. Other mutations such as L210Q in the E2 region of the Indian Ocean lineage could enhance transovarial transmission of CHIKV by *Aedes albopictus* [83]. These mutations together may account for the rapid spread of CHIKV by enhancing CHIKV prevalence in *Aedes albopictus* mosquitoes, a vector populating in Southeast Asia, and by facilitating viral spread to urban centers and regions with colder climates [84]. A T249P amino acid substitution in the NS3 helicase of North American WNV increased virulence in American crow, a major natural reservoir for WNV [85]. On the other hand, a V159A substitution in the envelope protein of NA/WN02 strain, which replaced the initial New York NY99 strain in 2002 as the prevalent WNV strain in North America, reduced the extrinsic incubation period in *Culex spp.* mosquitoes, facilitating WNV prevalence in mosquitoes. These mutations, when combined together, may contribute to WNV rapid spread and persistence in North America [86,87]. 

Genome-wide comparative analysis of the pre-epidemic ZIKV strains (before the year 2007) and recent epidemic strains revealed that the structural changes in the 3-terminal untranslated region (3′-UTR) stem-loop might increase ZIKV transmissibility and virulence [88]. Additionally, the amount and function of sfRNA are determined by point mutations in the 3′-UTR. In mosquitos infected with DENV-2, sfRNA accumulated in the salivary glands and increased efficient transmission to the host [89].

## 5. Conclusions and Perspectives

It is difficult to predict which arbovirus, and when and where it will resurge next because an outbreak depends on multiple factors. While factors such as climate, travel/transportation, human/vector population density, and vector competence are important, genetic changes in viruses are the most unpredictable element. These changes happen quickly and each season can present a new challenge. Development of vaccines has been hampered by immune interference between multiple circulating serotypes, genotype, limited animal models, and a lack of knowledge about immune responses to these viruses and the association between secondary infection and risk of severe diseases [92]. Although population genetics studies have revealed an association of many host factors/SNPs with infection outcomes, in-depth understanding of their mechanism of action and robust validation of these clinical results are still missing. This knowledge could allow us to take more precautious measures for those vulnerable populations. Similarly, functional validation of viral mutations in infectivity and disease pathogenesis following each epidemic will need significant research efforts and could help us develop broader and more efficacious vaccines and antiviral drugs. 

## Figures and Tables

**Table 1:** Human single nucleotide polymorphisms/mutations associated with increased risk for severe disease outcomes.

Cellular Pathway	Gene/Mutation	Outcome of Infection	Reference
Type I IFN response	*OAS1B*/C820T	Increased susceptibility to WNV infection	[21]
	*IRF3*, *MX1*/SNPs	Increased susceptibility to WNV infection	[23]
	*OAS1*/TT, rs34137742	Increased susceptibility to WNV infection	[23]
	*OAS3*/SNP, rs2285933	Increased dengue infection	[24]
Viral recognition	*TLR7*, *TLR8*/SNPs	Increased susceptibility to CHIKV infection	[25]
	CLEC5A-CT/TT (rs1285933)	Increased risk for DENV diseases and TNF levels	[26,27]
	CD209-336A/G	Associated with DHF P and correlated to DC-SIGN expression and immune augmentation	[29,30]
Immunoglobulin	*FCGR 2A*	Severe dengue infection	[31,32]
Blood type	Type A, RH+	Increased susceptibility to CHIKV infection	[34]
Neuron progenitor cell growth and differentiation	*MSI1*/wild type	Increasing ZIKV replication by binding to 3′-UTR in neural precursors	[35]
MHC-I/II, T cell activation	DQA1*01:02	Increased susceptibility to WNV infection	[37]
	HLA-A*24	Increased risk for DHF and DSS	[40]
	HLA-A*0207, HLA-B*51	Increased risk for DHF	[41]
	HLA-A*31, HLA-B*15	Symptomatic dengue infection	[42]
	DQB1*0302, DQB1*0202	Increased risk for DHF and DF	[43]
	HLA-A*31 and HLA-DRB1*08	Increased risk for DSS	[44]
	HLA-A*24 and HLA-DRB1*12	Increased risk for DHF	[44]
Chemokine, immune cell recruitment	*CCR5*/∆32	Increased susceptibility to WNV infection	[45]
Inflammatory cytokine, tumor necrosis alpha	*TNFA -308A*	Increased risk for DHF	[46,47,48,49]
	*TNFA-238A*, *-238GA*	Increased risk for DHF and DSS	[50]
Anti-inflammation, interleukin 10	*IL10* (-1082/-819/-592) ACC/ATA	Increased risk for DHF	As above
Tryptase	*TPSAB1*/homozygous alpha allele	Increased risk for DSS	[51]
Phospholipase	*PLCE1*	Increased risk for DSS	[52,53]

DHF: dengue hemorrhagic fever, DSS: dengue shock syndrome, UTR: untranslated region, *: Locus of SNP.

**Table 2:** Viral genetic mutations/polymorphisms that enhance infectivity, pathogenicity, fitness, and transmissibility.

Virus	Gene/Mutation	Outcome	Reference
DENV	NS2A	1998 DENV4 Puerto Rican epidemic strain	[61]
	E/T359A	1980 DENV2, subtype II Puerto Rican epidemic strain	[62]
	C/R97K-NS1/K94R-NS3/P245T	Asian/American to NI-1 clade	[63]
	NS4B/N245S	NI-1 to NI-2 clade	Same as above
	E/M492V-NS1/L279F NS5/K200Q, T290I, R401K	NI-2 to NI-2B clade	Same as above
	NS5	DENV2 -more pathogenic to human host	[64]
	prM/NS2A/NS4A	Attenuation of Togan strains	[65]
	3′-UTR/sfRNA polymorphism	Increased infectivity and pathogenicity in humans, transmission by mosquitoes	[66,67,89]
	NS5/A811V	Increased disease severity	[90]
ZIKV	E/V603I, D679E	Recently epidemic strains, unknown outcome	[70]
	PrM/V153M	Recently epidemic strains, unknown outcome	[70]
	prM/S139N	Increased infectivity in neural progenitor cells	[71]
	NS1/A188V	Enhanced NS1 secretability in the host blood, immune suppression in mosquitoes	[78]
CHIKV	E1/K211T	Increased fitness in *Ae. aegypti* mosquitoes	[79,81]
	E2/V264A	Increased fitness in *Ae. aegypti* mosquitoes	[80]
	E1/A226V	Increased fitness in *Ae. albopictus* mosquitoes, reduced dependence on cholesterol for replication	[82]
	E2/L210Q	Enhanced transovarial transmission by *Ae. albopictus*	[83]
WNV	NS3/T249P	Increased adaption to American crow	[85,91]
	E/V159A	Increased fitness in *Culex spp*. mosquitoes	[86,87]

sfRNA: subgenomic flaviviral RNA, DHF: dengue hemorrhagic fever, E: envelope, NS: nonstructural, UTR: untranslated genomic region.

## References

[1] Bryant J.E., Holmes E.C., Barrett A.D. (2007). Out of Africa: A molecular perspective on the introduction of yellow fever virus into the Americas. PLoS Pathog..

[2] Gubler D.J. (1998). Resurgent vector-borne diseases as a global health problem. Emerg. Infect. Dis..

[3] CDC Yellow Fever. https://www.cdc.gov/globalhealth/newsroom/topics/yellowfever/index.html.

[4] Brent S.E., Watts A., Cetron M., German M., Kraemer M.U., Bogoch I.I., Brady O.J., Hay S.I., Creatore M.I., Khan K. (2018). International travel between global urban centres vulnerable to yellow fever transmission. Bull. World Health Organ..

[5] Bhatt S., Gething P.W., Brady O.J., Messina J.P., Farlow A.W., Moyes C.L., Drake J.M., Brownstein J.S., Hoen A.G., Sankoh O. (2013). The global distribution and burden of dengue. Nature.

[6] Gubler D.J., Trent D.W. (1993). Emergence of epidemic dengue/dengue hemorrhagic fever as a public health problem in the Americas. Infect. Agents Dis..

[7] Gubler D.J., Campbell G.L., Nasci R., Komar N., Petersen L., Roehrig J.T. (2000). West Nile virus in the United States: Guidelines for detection, prevention, and control. Viral. Immunol..

[8] ECDC West Nile Fever. https://ecdc.europa.eu/en/news-events/epidemiological-update-west-nile-fever-europe-number-infections-so-far-exceeds-total.

[9] Shragai T., Tesla B., Murdock C., Harrington L.C. (2017). Zika and chikungunya: Mosquito-borne viruses in a changing world. Ann. N. Y. Acad. Sci..

[10] CDC Zika Cases in the United States. https://www.cdc.gov/zika/reporting/case-counts.html.

[11] Gubler D.J. (1998). Dengue and dengue hemorrhagic fever. Clin. Microbiol. Rev..

[12] Gubler D.J., Suharyono W., Lubis I., Eram S., Gunarso S. (1981). Epidemic dengue 3 in central Java, associated with low viremia in man. Am. J. Trop. Med. Hyg..

[13] Lee K.S., Lai Y.L., Lo S., Barkham T., Aw P., Ooi P.L., Tai J.C., Hibberd M., Johansson P., Khoo S.P. (2010). Dengue virus surveillance for early warning, Singapore. Emerg. Infect. Dis..

[14] Ooi E.E., Hart T.J., Tan H.C., Chan S.H. (2001). Dengue seroepidemiology in Singapore. Lancet.

[15] Ooi E.-E., Goh K.-T., Gubler D.J. (2006). Dengue prevention and 35 years of vector control in Singapore. Emerg. Infect. Dis..

[16] Ooi E.E., Gubler D.J. (2009). Dengue in Southeast Asia: Epidemiological characteristics and strategic challenges in disease prevention. Cad. Saude Publica.

[17] Guzman M.G., Halstead S.B., Artsob H., Buchy P., Farrar J., Gubler D.J., Hunsperger E., Kroeger A., Margolis H.S., Martínez E. (2010). Dengue: A continuing global threat. Nat. Rev. Microbiol..

[18] Schreiber M.J., Holmes E.C., Ong S.H., Soh H.S., Liu W., Tanner L., Aw P.P., Tan H.C., Ng L.C., Leo Y.S. (2009). Genomic epidemiology of a dengue virus epidemic in urban Singapore. J. Virol..

[19] Mashimo T., Lucas M., Simon-Chazottes D., Frenkiel M.P., Montagutelli X., Ceccaldi P.E., Deubel V., Guenet J.L., Despres P. (2002). A nonsense mutation in the gene encoding 2′-5′-oligoadenylate synthetase/L1 isoform is associated with West Nile virus susceptibility in laboratory mice. Proc. Natl. Acad. Sci. USA.

[20] Perelygin A.A., Scherbik S.V., Zhulin I.B., Stockman B.M., Li Y., Brinton M.A. (2002). Positional cloning of the murine flavivirus resistance gene. Proc. Natl. Acad. Sci. USA.

[21] Yakub I., Lillibridge K.M., Moran A., Gonzalez O.Y., Belmont J., Gibbs R.A., Tweardy D.J. (2005). Single nucleotide polymorphisms in genes for 2′-5′-oligoadenylate synthetase and RNase L inpatients hospitalized with West Nile virus infection. J. Infect. Dis..

[22] Lim J.K., Lisco A., McDermott D.H., Huynh L., Ward J.M., Johnson B., Johnson H., Pape J., Foster G.A., Krysztof D. (2009). Genetic variation in OAS1 is a risk factor for initial infection with West Nile virus in man. PLoS Pathog..

[23] Bigham A.W., Buckingham K.J., Husain S., Emond M.J., Bofferding K.M., Gildersleeve H., Rutherford A., Astakhova N.M., Perelygin A.A., Busch M.P. (2011). Host genetic risk factors for West Nile virus infection and disease progression. PLoS ONE.

[24] Simon-Loriere E., Lin R.J., Kalayanarooj S.M., Chuansumrit A., Casademont I., Lin S.Y., Yu H.P., Lert-Itthiporn W., Chaiyaratana W., Tangthawornchaikul N. (2015). High Anti-Dengue Virus Activity of the OAS Gene Family Is Associated With Increased Severity of Dengue. J. Infect. Dis..

[25] Dutta S.K., Tripathi A. (2017). Association of toll-like receptor polymorphisms with susceptibility to chikungunya virus infection. Virology.

[26] Xavier-Carvalho C., Gibson G., Brasil P., Ferreira R.X., de Souza Santos R., Goncalves Cruz O., de Oliveira S.A., de Sa Carvalho M., Pacheco A.G., Kubelka C.F. (2013). Single nucleotide polymorphisms in candidate genes and dengue severity in children: A case-control, functional and meta-analysis study. Infect. Genet. Evol..

[27] Xavier-Carvalho C., Cezar R., Freire N.M., Vasconcelos C.M.M., Solorzano V.E.F., de Toledo-Pinto T.G., Fialho L.G., do Carmo R.F., Vasconcelos L.R.S., Cordeiro M.T. (2017). Association of rs1285933 single nucleotide polymorphism in CLEC5A gene with dengue severity and its functional effects. Hum. Immunol..

[28] Chen S.T., Lin Y.L., Huang M.T., Wu M.F., Cheng S.C., Lei H.Y., Lee C.K., Chiou T.W., Wong C.H., Hsieh S.L. (2008). CLEC5A is critical for dengue-virus-induced lethal disease. Nature.

[29] Sakuntabhai A., Turbpaiboon C., Casademont I., Chuansumrit A., Lowhnoo T., Kajaste-Rudnitski A., Kalayanarooj S.M., Tangnararatchakit K., Tangthawornchaikul N., Vasanawathana S. (2005). A variant in the CD209 promoter is associated with severity of dengue disease. Nat. Genet..

[30] Wang L., Chen R.F., Liu J.W., Lee I.K., Lee C.P., Kuo H.C., Huang S.K., Yang K.D. (2011). DC-SIGN (CD209) Promoter -336 A/G polymorphism is associated with dengue hemorrhagic fever and correlated to DC-SIGN expression and immune augmentation. PLoS Negl. Trop. Dis..

[31] Mohsin S.N., Mahmood S., Amar A., Ghafoor F., Raza S.M., Saleem M. (2015). Association of FcgammaRIIa Polymorphism with Clinical Outcome of Dengue Infection: First Insight from Pakistan. Am. J. Trop. Med. Hyg..

[32] Garcia G., Sierra B., Perez A.B., Aguirre E., Rosado I., Gonzalez N., Izquierdo A., Pupo M., Danay Diaz D.R., Sanchez L. (2010). Asymptomatic dengue infection in a Cuban population confirms the protective role of the RR variant of the FcgammaRIIa polymorphism. Am. J. Trop. Med. Hyg..

[33] Noecker C.A., Amaya-Larios I.Y., Galeana-Hernandez M., Ramos-Castaneda J., Martinez-Vega R.A. (2014). Contrasting associations of polymorphisms in FcgammaRIIa and DC-SIGN with the clinical presentation of dengue infection in a Mexican population. Acta. Trop..

[34] Gallian P., Leparc-Goffart I., Richard P., Maire F., Flusin O., Djoudi R., Chiaroni J., Charrel R., Tiberghien P., de Lamballerie X. (2017). Epidemiology of Chikungunya Virus Outbreaks in Guadeloupe and Martinique, 2014: An Observational Study in Volunteer Blood Donors. PLoS Negl. Trop. Dis..

[35] Chavali P.L., Stojic L., Meredith L.W., Joseph N., Nahorski M.S., Sanford T.J., Sweeney T.R., Krishna B.A., Hosmillo M., Firth A.E. (2017). Neurodevelopmental protein Musashi-1 interacts with the Zika genome and promotes viral replication. Science.

[36] Weiskopf D., Angelo M.A., de Azeredo E.L., Sidney J., Greenbaum J.A., Fernando A.N., Broadwater A., Kolla R.V., De Silva A.D., de Silva A.M. (2013). Comprehensive analysis of dengue virus-specific responses supports an HLA-linked protective role for CD8+ T cells. Proc. Natl. Acad. Sci. USA.

[37] Sarri C.A., Markantoni M., Stamatis C., Papa A., Tsakris A., Pervanidou D., Baka A., Politis C., Billinis C., Hadjichristodoulou C. (2016). Genetic Contribution of MHC Class II Genes in Susceptibility to West Nile Virus Infection. PLoS ONE.

[38] Lanteri M.C., Kaidarova Z., Peterson T., Cate S., Custer B., Wu S., Agapova M., Law J.P., Bielawny T., Plummer F. (2011). Association between HLA class I and class II alleles and the outcome of West Nile virus infection: An exploratory study. PLoS ONE.

[39] Nguyen T.P., Kikuchi M., Vu T.Q., Do Q.H., Tran T.T., Vo D.T., Ha M.T., Vo V.T., Cao T.P., Tran V.D. (2008). Protective and enhancing HLA alleles, HLA-DRB1*0901 and HLA-A*24, for severe forms of dengue virus infection, dengue hemorrhagic fever and dengue shock syndrome. PLoS Negl. Trop. Dis..

[40] Loke H., Bethell D.B., Phuong C.X., Dung M., Schneider J., White N.J., Day N.P., Farrar J., Hill A.V. (2001). Strong HLA class I—Restricted T cell responses in dengue hemorrhagic fever: A double-edged sword?. J. Infect. Dis..

[41] Stephens H.A., Klaythong R., Sirikong M., Vaughn D.W., Green S., Kalayanarooj S., Endy T.P., Libraty D.H., Nisalak A., Innis B.L. (2002). HLA-A and -B allele associations with secondary dengue virus infections correlate with disease severity and the infecting viral serotype in ethnic Thais. Tissue Antigens.

[42] Sierra B., Alegre R., Perez A.B., Garcia G., Sturn-Ramirez K., Obasanjo O., Aguirre E., Alvarez M., Rodriguez-Roche R., Valdes L. (2007). HLA-A, -B, -C, and -DRB1 allele frequencies in Cuban individuals with antecedents of dengue 2 disease: Advantages of the Cuban population for HLA studies of dengue virus infection. Hum. Immunol..

[43] Falcon-Lezama J.A., Ramos C., Zuniga J., Juarez-Palma L., Rangel-Flores H., Garcia-Trejo A.R., Acunha-Alonzo V., Granados J., Vargas-Alarcon G. (2009). HLA class I and II polymorphisms in Mexican Mestizo patients with dengue fever. Acta Trop..

[44] Malavige G.N., Rostron T., Rohanachandra L.T., Jayaratne S.D., Fernando N., De Silva A.D., Liyanage M., Ogg G. (2011). HLA class I and class II associations in dengue viral infections in a Sri Lankan population. PLoS ONE.

[45] Glass W.G., Lim J.K., Cholera R., Pletnev A.G., Gao J.L., Murphy P.M. (2005). Chemokine receptor CCR5 promotes leukocyte trafficking to the brain and survival in West Nile virus infection. J. Exp. Med..

[46] Perez A.B., Sierra B., Garcia G., Aguirre E., Babel N., Alvarez M., Sanchez L., Valdes L., Volk H.D., Guzman M.G. (2010). Tumor necrosis factor-alpha, transforming growth factor-beta1, and interleukin-10 gene polymorphisms: Implication in protection or susceptibility to dengue hemorrhagic fever. Hum. Immunol..

[47] Fernando A.N., Malavige G.N., Perera K.L., Premawansa S., Ogg G.S., De Silva A.D. (2015). Polymorphisms of Transporter Associated with Antigen Presentation, Tumor Necrosis Factor-alpha and Interleukin-10 and their Implications for Protection and Susceptibility to Severe Forms of Dengue Fever in Patients in Sri Lanka. J. Glob. Infect. Dis..

[48] Fernandez-Mestre M.T., Gendzekhadze K., Rivas-Vetencourt P., Layrisse Z. (2004). TNF-alpha-308A allele, a possible severity risk factor of hemorrhagic manifestation in dengue fever patients. Tissue Antigens.

[49] Santos A.C., de Moura E.L., Ferreira J.M., Santos B.R., Alves V.M., de Farias K.F., de Souza Figueiredo E.V. (2017). Meta-Analysis of the Relationship between TNF-alpha (-308G/A) and IL-10 (-819C/T) Gene Polymorphisms and Susceptibility to Dengue. Immunol. Investig..

[50] Sam S.S., Teoh B.T., Chinna K., AbuBakar S. (2015). High producing tumor necrosis factor alpha gene alleles in protection against severe manifestations of dengue. Int. J. Med. Sci..

[51] Velasquez C.V., Roman A.D., Lan N.T., Huy N.T., Mercado E.S., Espino F.E., Perez M.L., Huong V.T., Thuy T.T., Tham V.D. (2015). Alpha tryptase allele of Tryptase 1 (TPSAB1) gene associated with Dengue Hemorrhagic Fever (DHF) and Dengue Shock Syndrome (DSS) in Vietnam and Philippines. Hum. Immunol..

[52] Dang T.N., Naka I., Sa-Ngasang A., Anantapreecha S., Chanama S., Wichukchinda N., Sawanpanyalert P., Patarapotikul J., Tsuchiya N., Ohashi J. (2014). A replication study confirms the association of GWAS-identified SNPs at MICB and PLCE1 in Thai patients with dengue shock syndrome. BMC Med. Genet..

[53] Khor C.C., Chau T.N., Pang J., Davila S., Long H.T., Ong R.T., Dunstan S.J., Wills B., Farrar J., Van Tram T. (2011). Genome-wide association study identifies susceptibility loci for dengue shock syndrome at MICB and PLCE1. Nat. Genet..

[54] Moya A., Holmes E.C., Gonzalez-Candelas F. (2004). The population genetics and evolutionary epidemiology of RNA viruses. Nat. Rev. Microbiol..

[55] Dolan P.T., Whitfield Z.J., Andino R. (2018). Mechanisms and Concepts in RNA Virus Population Dynamics and Evolution. Annu. Rev. Virol..

[56] Costa R.L., Voloch C.M., Schrago C.G. (2012). Comparative evolutionary epidemiology of dengue virus serotypes. Infect. Genet. Evol..

[57] Messer W.B., Gubler D.J., Harris E., Sivananthan K., de Silva A.M. (2003). Emergence and global spread of a dengue serotype 3, subtype III virus. Emerg. Infect. Dis..

[58] Gubler D.J., Kuno G., Sather G.E., Velez M., Oliver A. (1984). Mosquito cell cultures and specific monoclonal antibodies in surveillance for dengue viruses. Am. J. Trop. Med. Hyg..

[59] Messer W.B., Vitarana U.T., Sivananthan K., Elvtigala J., Preethimala L.D., Ramesh R., Withana N., Gubler D.J., De Silva A.M. (2002). Epidemiology of dengue in Sri Lanka before and after the emergence of epidemic dengue hemorrhagic fever. Am. J. Trop. Med. Hyg..

[60] Gubler D.J., Reed D., Rosen L., Hitchcock J.R. (1978). Epidemiologic, clinical, and virologic observations on dengue in the Kingdom of Tonga. Am. J. Trop. Med. Hyg..

[61] Bennett S.N., Holmes E.C., Chirivella M., Rodriguez D.M., Beltran M., Vorndam V., Gubler D.J., McMillan W.O. (2003). Selection-driven evolution of emergent dengue virus. Mol. Biol. Evol..

[62] Bennett S.N., Holmes E.C., Chirivella M., Rodriguez D.M., Beltran M., Vorndam V., Gubler D.J., McMillan W.O. (2006). Molecular evolution of dengue 2 virus in Puerto Rico: Positive selection in the viral envelope accompanies clade reintroduction. J. Gen. Virol..

[63] OhAinle M., Balmaseda A., Macalalad A.R., Tellez Y., Zody M.C., Saborío S., Nuñez A., Lennon N.J., Birren B.W., Gordon A. (2011). Dynamics of Dengue Disease Severity Determined by the Interplay Between Viral Genetics and Serotype-Specific Immunity. Sci. Transl. Med..

[64] Chen H.L., Lin S.R., Liu H.F., King C.C., Hsieh S.C., Wang W.K. (2008). Evolution of dengue virus type 2 during two consecutive outbreaks with an increase in severity in southern Taiwan in 2001–2002. Am. J. Trop. Med. Hyg..

[65] Steel A., Gubler D.J., Bennett S.N. (2010). Natural attenuation of dengue virus type-2 after a series of island outbreaks: A retrospective phylogenetic study of events in the South Pacific three decades ago. Virology.

[66] Chapman E.G., Costantino D.A., Rabe J.L., Moon S.L., Wilusz J., Nix J.C., Kieft J.S. (2014). The structural basis of pathogenic subgenomic flavivirus RNA (sfRNA) production. Science.

[67] Manokaran G., Finol E., Wang C., Gunaratne J., Bahl J., Ong E.Z., Tan H.C., Sessions O.M., Ward A.M., Gubler D.J. (2015). Dengue subgenomic RNA binds TRIM25 to inhibit interferon expression for epidemiological fitness. Science.

[68] Umareddy I., Tang K.F., Vasudevan S.G., Devi S., Hibberd M.L., Gu F. (2008). Dengue virus regulates type I interferon signalling in a strain-dependent manner in human cell lines. J. Gen. Virol..

[69] Pinheiro T.M., Mota M.T.O., Watanabe A.S.A., Biselli-Perico J.M., Drumond B.P., Ribeiro M.R., Vedovello D., Araujo J.P., Pimenta P.F.P., Chaves B.A. (2018). Viral immunogenicity determines epidemiological fitness in a cohort of DENV-1 infection in Brazil. PLoS Negl. Trop. Dis..

[70] Chavez J.H., Silva J.R., Amarilla A.A., Moraes Figueiredo L.T. (2010). Domain III peptides from flavivirus envelope protein are useful antigens for serologic diagnosis and targets for immunization. Biologicals.

[71] Yuan L., Huang X., Liu Z., Zhang F., Zhu X., Yu J., Ji X., Xu Y., Li G., Li C. (2017). A single nucleotide mutation in the prM protein of Zika virus contributes to fetal microcephaly. Science.

[72] Winkler G., Randolph V.B., Cleaves G.R., Ryan T.E., Stollar V. (1988). Evidence that the mature form of the flavivirus nonstructural protein NS1 is a dimer. Virology.

[73] Post P.R., Carvalho R., Galler R. (1991). Glycosylation and secretion of yellow fever virus nonstructural protein NS1. Virus Res..

[74] Fan W.F., Mason P.W. (1990). Membrane association and secretion of the Japanese encephalitis virus NS1 protein from cells expressing NS1 cDNA. Virology.

[75] Crooks A.J., Lee J.M., Easterbrook L.M., Timofeev A.V., Stephenson J.R. (1994). The NS1 protein of tick-borne encephalitis virus forms multimeric species upon secretion from the host cell. J. Gen. Virol..

[76] Liu J., Liu Y., Nie K., Du S., Qiu J., Pang X., Wang P., Cheng G. (2016). Flavivirus NS1 protein in infected host sera enhances viral acquisition by mosquitoes. Nat. Microbiol..

[77] Alcon S., Talarmin A., Debruyne M., Falconar A., Deubel V., Flamand M. (2002). Enzyme-linked immunosorbent assay specific to Dengue virus type 1 nonstructural protein NS1 reveals circulation of the antigen in the blood during the acute phase of disease in patients experiencing primary or secondary infections. J. Clin. Microbiol..

[78] Liu Y., Liu J., Du S., Shan C., Nie K., Zhang R., Li X.F., Zhang R., Wang T., Qin C.F. (2017). Evolutionary enhancement of Zika virus infectivity in Aedes aegypti mosquitoes. Nature.

[79] Sumathy K., Ella K.M. (2012). Genetic diversity of Chikungunya virus, India 2006–2010: Evolutionary dynamics and serotype analyses. J. Med. Virol..

[80] Agarwal A., Sharma A.K., Sukumaran D., Parida M., Dash P.K. (2016). Two novel epistatic mutations (E1:K211E and E2:V264A) in structural proteins of Chikungunya virus enhance fitness in Aedes aegypti. Virology.

[81] Souza T.M., Azeredo E.L., Badolato-Correa J., Damasco P.V., Santos C., Petitinga-Paiva F., Nunes P.C., Barbosa L.S., Cipitelli M.C., Chouin-Carneiro T. (2017). First Report of the East-Central South African Genotype of Chikungunya Virus in Rio de Janeiro, Brazil. PLoS Curr..

[82] Schuffenecker I., Iteman I., Michault A., Murri S., Frangeul L., Vaney M.C., Lavenir R., Pardigon N., Reynes J.M., Pettinelli F. (2006). Genome microevolution of chikungunya viruses causing the Indian Ocean outbreak. PLoS Med..

[83] Niyas K.P., Abraham R., Unnikrishnan R.N., Mathew T., Nair S., Manakkadan A., Issac A., Sreekumar E. (2010). Molecular characterization of Chikungunya virus isolates from clinical samples and adult Aedes albopictus mosquitoes emerged from larvae from Kerala, South India. Virol. J..

[84] Tsetsarkin K.A., Vanlandingham D.L., McGee C.E., Higgs S. (2007). A single mutation in chikungunya virus affects vector specificity and epidemic potential. PLoS Pathog..

[85] Brault A.C., Huang C.Y., Langevin S.A., Kinney R.M., Bowen R.A., Ramey W.N., Panella N.A., Holmes E.C., Powers A.M., Miller B.R. (2007). A single positively selected West Nile viral mutation confers increased virogenesis in American crows. Nat. Genet..

[86] Ebel G.D., Carricaburu J., Young D., Bernard K.A., Kramer L.D. (2004). Genetic and phenotypic variation of West Nile virus in New York, 2000–2003. Am. J. Trop. Med. Hyg..

[87] Moudy R.M., Meola M.A., Morin L.L., Ebel G.D., Kramer L.D. (2007). A newly emergent genotype of West Nile virus is transmitted earlier and more efficiently by Culex mosquitoes. Am. J. Trop. Med. Hyg..

[88] Zhu Z., Chan J.F., Tee K.M., Choi G.K., Lau S.K., Woo P.C., Tse H., Yuen K.Y. (2016). Comparative genomic analysis of pre-epidemic and epidemic Zika virus strains for virological factors potentially associated with the rapidly expanding epidemic. Emerg. Microbes. Infect..

[89] Pompon J., Manuel M., Ng G.K., Wong B., Shan C., Manokaran G., Soto-Acosta R., Bradrick S.S., Ooi E.E., Misse D. (2017). Dengue subgenomic flaviviral RNA disrupts immunity in mosquito salivary glands to increase virus transmission. PLoS Pathog..

[90] Williams M., Mayer S.V., Johnson W.L., Chen R., Volkova E., Vilcarromero S., Widen S.G., Wood T.G., Suarez-Ognio L., Long K.C. (2014). Lineage II of Southeast Asian/American DENV-2 is associated with a severe dengue outbreak in the Peruvian Amazon. Am. J. Trop. Med. Hyg..

[91] Modis Y., Ogata S., Clements D., Harrison S.C. (2004). Structure of the dengue virus envelope protein after membrane fusion. Nature.

[92] McArthur M.A., Sztein M.B., Edelman R. (2013). Dengue vaccines: Recent developments, ongoing challenges and current candidates. Expert Rev. Vaccines.

